# A study on the prevalence of *Aeromonas* spp. and its enterotoxin genes in samples of well water, tap water, and bottled water

**DOI:** 10.14202/vetworld.2015.1237-1242

**Published:** 2015-10-28

**Authors:** Hareesh Didugu, Madhavarao Thirtham, Krishnaiah Nelapati, K Kondal Reddy, Baba Saheb Kumbhar, Anusha Poluru, Guruvishnu Pothanaboyina

**Affiliations:** 1 Animal Disease Diagnostic Laboratory, Vijayawada, Andhra Pradesh, India; 2 Department of Veterinary Public Health and Epidemiology, College of Veterinary Science, Proddatur, Andhra Pradesh, India; 3 Department of Veterinary Public Health, College of Veterinary Science, Rajendranagar, Telangana, India; 4 Department of Livestock Products Technology, Sri P.V. Narsimha Rao Telangana State University for Veterinary, Animal and Fishery Sciences, Rajendranagar, Telangana, India; 5 Department of Animal Genetics and Breeding, College of Veterinary Science, Proddatur, Andhra Pradesh, India

**Keywords:** *Aeromonas* spp, enterotoxins, polymerase chain reaction, prevalence, water

## Abstract

**Aim:**

The aim of this work was to study the prevalence of *Aeromonas* spp. and its enterotoxin genes in various water sources.

**Materials and Methods:**

125 samples (50 from well water, 50 from tap water, and 25 from bottled water) were collected from various sources in and around Greater Hyderabad Municipal Corporation and examined for the presence of aeromonads by both cultural and polymerase chain reaction (PCR) assay. Alkaline peptone water with ampicillin was used as enrichment. *Aeromonas* isolation medium and ampicillin dextrin agar were used as selective media. The boiling and snap chilling method was used for DNA extraction. Primers targeted against 16S rRNA, *aer*, and *ast* were used to identify aeromonads and its enterotoxins.

**Results:**

48%, 18%, and 12% of well water, tap water, and bottled water samples were found positive by cultural assay with an overall prevalence of 28.8%. Aeromonads were detected in 32 % (52% in well water, 20% in tap water, and 16% in bottled water) of samples by PCR assay. Aerolysin (*aer*) gene was noticed in 34.6%, 20%, and 0% of well water, tap water, and bottled water samples, respectively, with an overall prevalence of 27.5%. Thermostable cytotonic enterotoxin (*ast*) was observed in 37.5% (42.3% in well water, 30% in tap water, and 25% in bottled mineral water) of samples.

**Conclusions:**

Presence of aeromonads and its toxin genes in various sources of water is of public health concern and emphasizes the need for necessary preventive measures to tackle the problem.

## Introduction

*Aeromonas* spp. are ubiquitous in aquatic environments and was reported to be isolated from ground, surface, marine, drinking, and waste waters [1]. Some species of aeromonads were reported to be the cause of various diseases in aquatic animals, livestock, and humans. In 1968, Von Graevenitz and Mensch reported the importance of *Aeromonas* spp. as a human pathogen and suggested aeromonads may be associated with gastrointestinal disease [2]. Today aeromonads were reported to be the cause of community acquired infection, nosocomial infection, and travelers’ diarrhea and infections associated with hurricanes, tsunamis, and earthquakes [3].

The ability of aeromonads to colonize drinking water systems, produce biofilms and resist chlorination is of public health significance, as the organisms are able to elaborate toxins and cause various disease manifestations ranging from gastroenteritis to septicemia. Hence, considering its importance *Aeromonas hydrophila* was listed in the contaminant candidate list [4] and the Environmental Protection Agency Method 1605 was validated for its detection and enumeration in drinking water system. In 1986, health authorities in the Netherlands introduced “indicative maximum values” for *Aeromonas* densities in drinking-water [5]. Most important hemolysin produced by *Aeromonas* spp. is aerolysin (also called as cytotoxic enterotoxin, asao toxin and cholera toxin cross-reactive cytolytic enterotoxin), which possess both hemolytic and enterotoxic properties [4]. Environmental strains containing aerolysin are potentially enterotoxigenic when passed from host to host, but environmentally adapted strains are not pathogenic when acquired directly from the environment [4]. The gene *ast* is a thermostable cytotonic enterotoxin, which causes fluid accumulation in ligated ileal loops in animal models and probably has an undescribed role in causing diarrhea in humans [6]. Aeromonads are able to grow in water with a wide variation in temperature ranging from 0°C to 45°C, with an optimum of 22-32°C.

Most of the infections caused by *Aeromonas* spp. are acquired via contact with contaminated water sources or through ingestion of foods in various “farm to table” operations [7]. Khajanchi *et al*. [8] reported water and clinical isolates were found to have the same virulence signature, suggesting transmission of *Aeromonas* spp. from water to humans. In this regard, a study was conducted to investigate the prevalence of emerging pathogen - *Aeromonas* spp. and its enterotoxin genes in various water sources.

## Materials and Methods

### Ethical approval

Live animals were not used in this study, so ethical approval was not necessary. Water samples were collected from various water sources.

### Sample collection

125 samples (50 from well water, 50 from tap water, and 25 from bottled water) were collected under aseptic conditions and transferred back to the laboratory at the earliest possible. Well water samples were collected from various artesian wells, dug wells, and bore wells. Tap water samples were collected from domestic water supply lines. Bottled water samples were collected from water tins supplied to households and bottled water from various brands available in the market. All the samples were collected in and around Greater Hyderabad Municipal Corporation, Telangana, India.

### Conventional method

**Figure-1:**
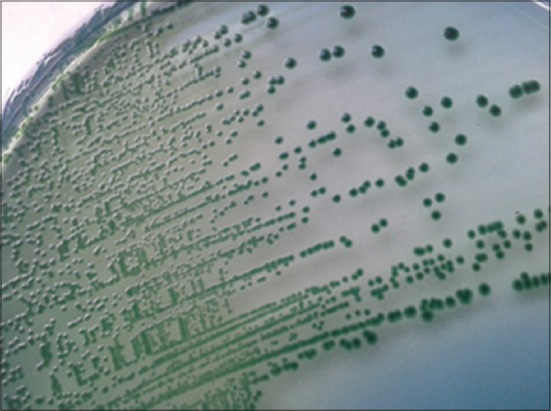
*Aeromonas* isolation medium displaying *Aeromonas* spp.

**Figure-2:**
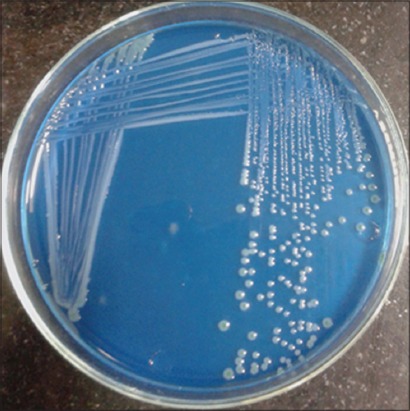
Ampicillin dextrin agar displaying *Aeromonas* spp.

10 ml of sample was inoculated into 90 ml of alkaline peptone water with ampicillin (APW-A) 10 mg/L and incubated at 37°C for 18 h. The enriched inoculum from APW-A was streaked on to *Aeromonas* isolation medium (Figure-1) and ampicillin dextrin agar (ADA) (Figure-2) and incubated at 37°C for 24 h. The presumptive colonies were streaked on nutrient agar and subjected to biochemical tests for confirmation [9] (Table-1). All the media were obtained from Himedia^®^ labs, India.

**Table-1:** List of biochemical tests conducted for confirmation of *Aeromonas* spp.

Tests	Typical reactions of *Aeromonas*
Gram’s reaction	Negative
Oxidase	Positive
Morphology	Coccobacilli
Motility	Positive
Catalase	Positive
0/129 vibriostatic agent (150 µg/g)	Resistant
Acid from glucose	Positive
Mannitol to acid	Positive
Lysine decarboxylase	Positive
Arginine decarboxylase	Negative
Growth on TSI	Acid butt, acid or alkaline slant, H_2_S negative, positive or negative for gas production
ONPG	Positive
Citrate	Positive

ONPG=Orthonitrophenyl beta-D-galactopyranoside, TSI=Triple sugar iron

### Polymerase chain reaction (PCR) assay

Bacterial DNA was obtained by boiling and snap chilling protocol [10]. The sample was inoculated into APW-A, and 1.5 ml of incubated broth was taken in a micro centrifuge tube. The tube was then centrifuged at 8000 rpm for 10 min, and the supernatant was discarded. 50 μl of sterile distilled water was added to the tubes and boiled in a water bath at 90°C for 10 min and immediately transferred onto ice. Further, the tube was centrifuged at 13,000 rpm for 5 min. For PCR assay, 2 µl of the bacterial lysate was taken as a template.

The primers [11,12] used for the detection of *Aeromonas* spp. and its toxins were custom synthesized by SR life science solutions^®^ (Table-2). Master mix was prepared by using 2 µl of the bacterial lysate, 2 µl of ×10 Taq polymerase buffer, 1.2 µl of Mgcl_2_, 1 µl of Taq DNA polymerase (1 U/μl), 0.8 µl of 10 mM dNTP mix, and 2 µl each of forward and reverse primer (10 pmol/µl), which was made up to 20 µl using molecular grade water. Cycling conditions followed for various primers were mentioned in Table-3. Routinely, a master mix was set up for 18 µl and distributed to the PCR tubes, to which 2 µl of the template was added. The samples were analyzed in 1.5% agarose gel electrophoresis with ethidium bromide. Two *Aeromonas* spp. *viz. A. hydrophila* (MTCC 1739) and *Aeromonas sobria* (MTCC 3613) were obtained from MTCC (microbial type culture collection), Chandigarh. The results obtained by both cultural and PCR assay were compared with the standard cultures and confirmed the presence of *Aeromonas* spp.

**Table-2:** List of primers used for detection of *Aeromonas* spp. and its toxins.

Primer	Target gene	Length		Primer sequence	Amplification product (bp)	Reference
16S rRNA	16S rRNA	21	5’F	TCA TGG CTC AGA TTG AAC GCT	599	11
		24	5’R	CGG GGC TTT CAC ATC TAA CTT ATC		
Aerolysin	*aer*	18	5’F	GCA GAA CCC ATC TAT CCA G	252	11
		20	5’R	TTT CTC CGG TAA CAG GAT TG		
Cytotonic enterotoxin	*ast*	21	5’F	TCT CCA TGC TTC CCT TCC ACT	331	12
		21	5’R	GTG TAG GGA TTG AAG AAG CCG		

**Table-3:** Thermal cycling conditions followed for various genes.

Step	16S rRNA	*aer*	*ast*
Initial denaturation	94°C/5 min	94°C/5 min	95°C/5 min
Final denaturation	94°C/1 min 30 cycles	94°C/1 min 30 cycles	95°C/25 s 25 cycles
Annealing	55°C/1 min	55°C/1 min	55°C/30 s
Initial extension	72°C/1 min	72°C/1 min	72°C/1 min
Final extension	72°C/5 min	72°C/5 min	70°C/5 min
Hold	4°C	4°C	4°C

## Results and Discussion

Among 125 samples investigated, 48%, 18%, and 12% samples of well water, tap water, and bottled water were found positive by the cultural method, respectively. 26 (52%) samples in well water, 10 (20%) in tap water, and 4 (16%) in bottled water were found positive by PCR assay, targeting 16S rRNA. Aerolysin was detected in well water (34.6%) and tap water (20%) with an overall prevalence of 15% among the isolates positive by PCR. 42.3%, 30%, and 25% of samples positive by PCR revealed the presence of thermostable cytotonic enterotoxin in well, tap, and bottled water samples, respectively. All the results were presented in detail in Table-4.

**Table-4:** Results of *Aeromonas* spp. and its toxins obtained from well, tap, and bottled water sources.

Type of sample	No. of samples	Positive result for *Aeromonas* spp.** (N (%))		% of cultural method compared to PCR	Distribution of toxins among isolates positive by PCR (N (%))	
	
		Cultural method	PCR assay		*aer*	*ast*
Well water	50	24 (48)	26 (52)	92.3	9 (34.6)	11 (42.3)
Tap water	50	9 (18)	10 (20)	90.0	2 (20.0)	3 (30.0)
Bottled water	25	3 (12)	4 (16)	75.0	0 (0.0)	1 (25.0)
Total	125	36 (28.8)	40 (32)	90.0	11 (27.5)	15 (37.5)

PCR=Polymerase chain reaction

**Figure-3:**
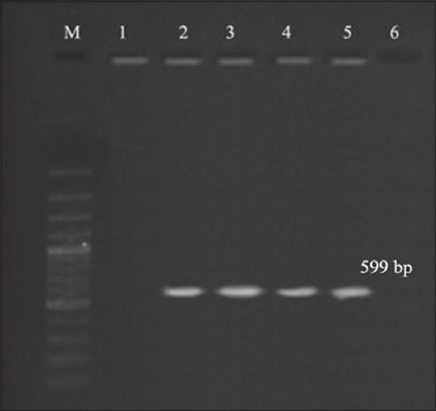
Polymerase chain reaction bands of *Aeromonas* spp. isolates form different water samples, Lane M: 100 bp DNA ladder, Lane 2, 3: Well water samples, Lane 4: Tap water samples, Lane 5: Bottled water samples.

Prevalence of 48% and 52% reported by cultural and PCR assays, respectively (Figure-3), in well water samples in this study were in agreement with the results of 48.7% in Libya [13] and 50% in Palestina [14], whereas lower prevalence of 8.3% [15] and 22.5% [16] were reported. Contrary to the findings of this study higher prevalence of 94.8% in various water sources in Norway [17] and cent percent in fresh water samples from Cambe stream, Brazil [18] were also reported.

Tap water is a common source of drinking water in urban areas. Among the Tap water samples examined, aeromonads were detected in 18% of samples by the cultural method and 20% of samples by PCR assay (Figure-3). These findings were in agreement with the results of 16% by Eid *et al*. [19] in Egypt. Pablos *et al*. [20] analyzed drinking-water samples in Spain and found 26.5% were positive for aeromonads. On the contrary, lower prevalence were reported by many authors [15,21-23], which might be due to water treatment, chlorination and maintenance of supply lines in a proper manner. Prevalence of 46.2% in Palestina [14] and 96.3% in Chennai [23] were also reported indicating a high prevalence of aeromonads. The occurrence of *Aeromonas* spp. in chlorinated drinking water [24] and in drinking water reservoirs [25] were reported emphasizing the ability to resist chlorination.

**Figure-4:**
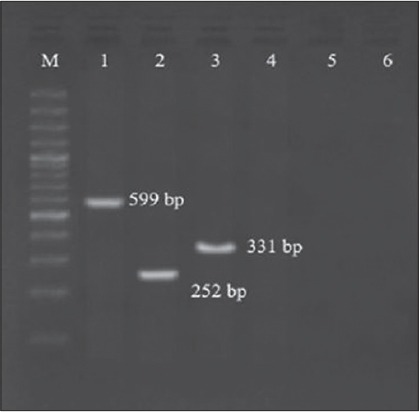
Comparison between amplicon products obtained from genes 16S rRNA, *aer*, and *ast* of *Aeromonas* spp. Lane M: 100 bp DNA ladder, Lane 1: Amplicon product of 16S rRNA, Lane 2: Amplicon product of *aer*., Lane 3: Amplicon product of *ast*.

Prevalence of 12% and 16% by cultural and PCR assay, respectively (Figure-3), were observed in bottled water samples in this study, which were similar to the findings of Scoaris *et al*. [15], whereas lower prevalence of 0% [26] was also reported. Biscardi *et al*. [27] reported the presence of aerolysin by isolating 6 strains of *A. hydrophila* from 61 mineral and thermal water samples. Higher prevalence of 43% in both bottled mineral water and municipal supply sources [28] and 75% in bottled water [29] were also reported. Day to day, there is increasing concern among people regarding health and hygiene, leading to profound raise in consumption of bottled water since last decade. Hence, detection of *Aeromonas* spp. in bottled water should not be ignored, and there is a need for strict implementation of Hazard Analysis and Critical Control Points in the production of bottled water. Variation in the results among various authors may be due to differences in the site of sampling, time of sampling, source of sample, processing method adopted, geographic, seasonal variations, and procedures adopted for isolation [30]. In this study, in agreement with Gugliandolo *et al*. [31] and Liu [32], it is revealed that PCR (Figure-4) was a better, sensitive, and cheap method compared to cultural assay (90% compared to PCR). Balakrishna *et al*. [21] and Venkataiah *et al*. [9] suggested PCR as a better alternative, even though similar results were obtained by both conventional and PCR assay.

**Figure-5:**
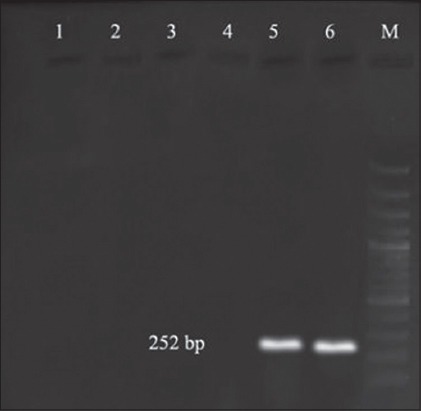
Polymerase chain reaction bands of *aer* gene from different water samples, Lane M: 100 bp DNA ladder, Lane 5: Well water samples showing positive results, Lane 6: Tap water samples showing positive results.

Prevalence of 34.6%, 20%, and 0% of aerolysin (*aer*) were observed in well water, tap water, and bottled water, respectively, with an overall prevalence of 27.5% in this study (Figure-5), whereas higher prevalence of 66.6% [33], 80.6% [16], and 88.9% [10] were reported in various environmental samples. Ormen and Ostensvik [17] reported that 79% of isolates carry aerolysin gene in ambient water and drinking water in Norway. 38.5% of environmental samples carried *aer* gene and 44.8% contained at least one of the putative virulence properties [34]. The occurrence of aerolysin in mineral and thermal waters was also reported [27]. Even though all species of aeromonads may not contain toxin genes, high levels of prevalence noticed in the present study indicate the wide presence of aerolysin gene among the isolates in the studied area.

**Figure-6:**
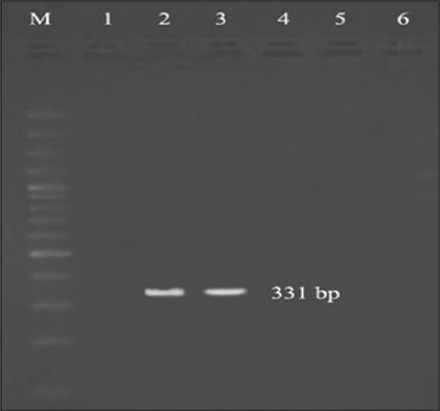
Polymerase chain reaction bands of *ast* gene from different water samples, Lane M: 100 bp DNA ladder, Lane 2: Well water samples showing positive results, Lane 3: Bottled water samples showing positive results.

An overall prevalence of 37.5% for *ast* gene (42.3%, 30%, and 25% from well water, tap water, and bottled water, respectively) was observed in this study (Figure-6), which was comparable to the results of various authors [12,34]. On contrary higher levels of prevalence (96.7% [16] and 97.6% [35]) were reported in variety of food and environmental samples, whereas Bhowmik *et al*. [26] reported that none among the surface waters examined were positive for *ast* gene. The degree of variation observed may be due differences in the expression of genes associated with environmental conditions [34].

A wide range of disease manifestations caused by *Aeromonas* spp. such as skin and soft tissue infection following Tsunami in Thailand, hurricane Katrina; cellulitis, myositis and septicemia in humans working in aquatic environment; gastrointestinal infection and acute renal failure from aquarium water; respiratory infections and pneumonia in accidental drowning were reported [4,7]. The presence of significant percentage of aeromonads and its enterotoxin genes in various samples of water in this study may lead to any of the above conditions, causing various human health complications. Hence, the versatility of aeromonads in varied ecosystems is of emphasis and the sobriquet “Jack of all trades” [36], by which aeromonads are called, cannot be ignored.

## Conclusion

From this study, it is concluded that aeromonads are prevalent in various water sources. Presence of toxin gene markers in isolates revealed the pathogenic potential of *Aeromonas* spp., emphasizing the importance of hygiene and proper monitoring at various stages of water treatment to reduce or to eliminate the risk of water borne Aeromoniasis.

## Authors’ Contributions

MT and KN designed and planned the research experiments. KRK suggested and supervised. HD performed the research work and drafted manuscript. BSNK and AP helped in conducting research. GVP helped in PCR standardization.
